# Effects of l‐arginine supplementation in patients with sickle cell disease: A systematic review and meta‐analysis of clinical trials

**DOI:** 10.1002/hsr2.1167

**Published:** 2023-04-11

**Authors:** Alireza Sadeghi, Ehsan Taherifard, Niloofar Dehdari Ebrahimi, Elham Rafiei, Farshad Hadianfard, Erfan Taherifard

**Affiliations:** ^1^ Student Research Committee Shiraz University of Medical Sciences Shiraz Iran; ^2^ Internal Medicine Department Shiraz University of Medical Sciences Shiraz Iran

**Keywords:** arginine, meta‐analysis, sickle cell disease

## Abstract

**Background and Aims:**

Previous studies have shown that supplementation of some amino acids such as l‐arginine or its precursors could exert beneficial effects in patients with sickle cell disease (SCD). The objective of this study is to systematically review the literature to assess the effect of arginine administration on the clinical and paraclinical parameters of patients with SCD.

**Methods:**

Four online databases of PubMed, Web of Sciences, Scopus, and Embase were selected for systematic search. Eligible studies were clinical trials that evaluated the effect of arginine usage in patients with SCD. Effects sizes were calculated using weighted mean difference (WMD) and Hedge's g and they were pooled using random‐effects modeling with Hartung–Knapp adjustment. Additional analyses were also conducted.

**Results:**

Twelve studies containing detail of 399 patients with SCD were found to be eligible. The data synthesis showed that l‐arginine significantly increased the level of NO metabolites (Hedge's g: 1.50, 0.48–1.82, *I* ^2^: 88%) and hemoglobin F (WMD: 1.69%, 0.86–2.52, *I* ^2^: 0%) and significantly decreased systolic blood pressure (WMD: −8.46 mmHg, −15.58 to −1.33, *I* ^2^: 53%) and aspartate transaminase (Hedge's g: −0.49, −0.73 to −0.26, *I* ^2^: 0%). However, there were no significant effects on hemoglobin, reticulocyte, malondialdehyde and diastolic blood pressure, and alanine transaminase.

**Conclusion:**

Our meta‐analysis showed that l‐arginine use for SCD could be beneficial, increase hemoglobin F and exert blood pressure‐lowering and hepatoprotective properties. However, for a firm conclusion and widespread use of l‐arginine for these patients, more studies are needed.

## 1 INTRODUCTION

Sickle cell disease (SCD), a group of multisystem autosomally recessive inherited hemoglobin disorders, is caused by a point mutation in the gene encoding β chains of hemoglobin.^1^, ^2^ Although there is no accurate estimate of the global prevalence of SCD, it has been reported that nearly 6 million neonates are born each year with SCD, more than 300,000 with the homozygous genotype of the disease, worldwide.^3^ The mutated gene gives rise to the production of dysfunctional chains and therefore, the formation of a dysfunctional hemoglobin, HbS. This hemoglobin possesses a lower affinity for oxygen and is liable to polymerization upon response to some triggers such as low oxygen tension, imbalance of acid–base homeostasis, and alteration in the temperature which results in highly inflexible, fragile, and adhesive erythrocytes.^4^ These abnormal erythrocytes contribute to the main features of this multisystem disorder, premature destruction of the red cells, hemolysis and the ensuing anemia, and vaso‐occlusion.^5^ Substances such as arginase 1 and asymmetric dimethylarginine which are released from ruptured red cells target the synthesis of nitric oxide (NO) and its downstream pathway which has an important role in the pathophysiology of the course of SCD.^1^, ^6^

Previous studies have shown that supplementation of some amino acids such as l‐arginine or its precursors could exert beneficial effects in patients with SCD, during both the steady state and the crisis.^7^, ^8^, ^9^, ^10^ This amino acid is considered to be a conditionally essential amino acid, particularly under certain circumstances in patients with a catabolic state or in the presence of an acute stressor.^11^ l‐arginine contains a nitrogenous group of guanidine which involves binding to NO synthase and serves as a substrate for this enzyme for NO production.^11^, ^12^ In a randomized placebo‐controlled clinical trial, participants in the intervention group received l‐arginine at a dose of 500 mg daily for over 4 months.^13^ The study showed a significant increase in the level of NO metabolites in those on l‐arginine compared with patients in the placebo group. Besides, this study demonstrated that this dietary supplement provided an analgesic effect. In addition to these effects, it has also been reported that l‐arginine could have antioxidant, cardioprotective, hepatoprotective, and immunomodulatory properties and improve blood pressure control, metabolic profile components, and response to the therapeutic effects of hydroxyurea.^7^, ^14^, ^15^, ^16^, ^17^, ^18^

Despite this body of evidence regarding the beneficial effects, safety, and tolerability of supplemental arginine in patients with SCD, no consensus has been reached on its use; besides, no meta‐analysis has been conducted on the issue. The objective of this study is to systematically review the effect of l‐arginine administration on the clinical and paraclinical parameters of patients with SCD.

## 2 MATERIALS AND METHODS

### 2.1 Databases and search strategy

The protocol of this study was registered in the International Prospective Register of Systematic Reviews (PROSPERO) with the registration number CRD42022355733. In this review, we used Preferred Reporting Items for Systematic reviews and Meta‐Analysis (PRISMA) guideline version 2020.^19^

For the literature review, four online databases were selected, PubMed, Web of Sciences, Scopus, and Embase. For the search strategy, we used two groups of words related to arginine and SCD. The words for arginine were “Arginine”, “l‐arginine”, “ArginMax”, “AAKG”, “Argicap”, and “Argitall”. The words considered for SCD were “Sickle cell”, “SCD”, “Sickle cell syndrome”, “Sickle cell anemia”, “hemoglobin S disease”, “HBS disease”, and “Sickling disorder”. A combination of these terms was searched in the title, abstract, and keywords of the articles. We conducted our search twice; our primary search was done on April 24, 2022, and then was updated on January 31, 2023. The search strategies that we used in the online databases are provided in the Supplementary material. Furthermore, we checked the reference list and citations of records considered eligible to identify additional relevant documents. Respective main articles of eligible conference proceedings/abstracts were searched manually to retrieve the complete data.

### 2.2 Eligibility criteria

We restricted our research to trial studies that evaluated the effect of supplemental l‐arginine in patients with SCD. The eligible trials were on human subjects with an established diagnosis of SCD. We excluded studies in which a combination of arginine with other amino acids or supplements was administrated to the patients. Single‐arm studies, studies with a control group consisting of normal healthy individuals, nonrandomized trials, and noninferiority trials were not excluded from the study. There was no restriction on the age of the participants, the genotype of SCD (homozygous form or others), the setting of the patients (outpatient or inpatient), language, country, or publication year. Conference proceedings/abstracts, editorial papers, letters to the editor, and correspondences were removed in the screening rounds in the case that they presented the same findings; otherwise, they were included in the case that they met other eligibility criteria.

### 2.3 Selection process and data extraction

Two authors (A. S. and N. D. E.) conducted this systematic search. The records found upon the search in the databases were exported to Endnote library (Clarivate Analytics) version X9. After the removal of duplicate records, these two authors completed the first round of screening based on the titles and abstracts of the identified records. Then, full texts of the remaining records were retrieved and screened by two authors (A. S. and N. D. E.), independently. Disagreements between the authors were resolved by discussion. Interrater reliability was measured by both percent agreement and Cohen's *Κ*.^20^, ^21^

We designed an Excel spreadsheet for data extraction. These authors (Ehsan T. and Erfan T.) were responsible for the process. The following data were extracted from each study: first author, publication year, country, study design, the sample size in each group of the study, characteristics of the participants, percentage of males, age of the participants, the genotypic subtype of SCD, dosage, route and duration of administration of arginine and endpoints of each study and adverse events occurred in the course of study.

### 2.4 Risk of bias assessment

Based on the design of the included clinical trial, we used Cochrane tools for risk of bias assessment, revised Cochrane risk of bias tool for randomized trials (RoB2), and risk of bias in nonrandomized studies of interventions (ROBINS‐I).^22^, ^23^ The risk assessment was conducted by two authors (F. H. and E. R.), separately.

### 2.5 Certainty of evidence assessment

Assessment of certainty of the evidence was done by two authors (F. H. and E. R.) using the Grading of Recommendations, Assessment, Development and Evaluation (GRADE) approach, separately.^24^, ^25^ Using this approach, the quality of a body of evidence for each outcome was rated as one of the following four grades: high, moderate, low, or very low. This rating was determined by assessing the outcomes and its quantitative analysis against several factors including the presence of risk of bias, inconsistency of the results, indirectness of the evidence, imprecision, publication bias, large magnitude of an effect, dose–response gradient and effect of plausible residual confounding.

### 2.6 Statistical analysis

Data were analyzed using Comprehensive Meta‐Analysis (Biostat Inc.) version 3 and Stata (StataCorp LLC) version 17. We used the recommendations provided by Assel et al. for reporting the statistics in our study.^26^ The effects sizes of arginine use on NO metabolites concentration, hemoglobin, hemoglobin F (HbF), reticulocyte, malondialdehyde level, blood pressure level, both systolic and diastolic blood pressure (SBP and DBP), aspartate transaminase (AST), and alanine transaminase (ALT) were estimated. These effects were pooled using weighted mean difference (WMD) for hemoglobin, Hb, SBP, and DBP. For the rest of the variables, Hedge's g statistic was used. Then, random effects modeling, the DerSimonian–Laird model with standard‐error adjustment for the overall effect size of Hartung–Knapp, was applied.^27^ Standard deviations (SD) were measured from standard errors of the mean or confidence intervals (CI) by use of appropriate formulas when they were not reported in the articles.^28^ The *r* value as a correlation coefficient was measured using pre‐ and postadministration of arginine SDs and SD of change when available. Interstudy heterogeneity was assessed using *I* ^2^ statistics; we also calculated the 95% CIs for *I* ^2^.^29^ Sensitivity analyses and subgroup analyses were also performed. We used Egger's test for the presence of potential publication bias.

## 3 RESULTS

### 3.1 Literature search

**Figure 1:**
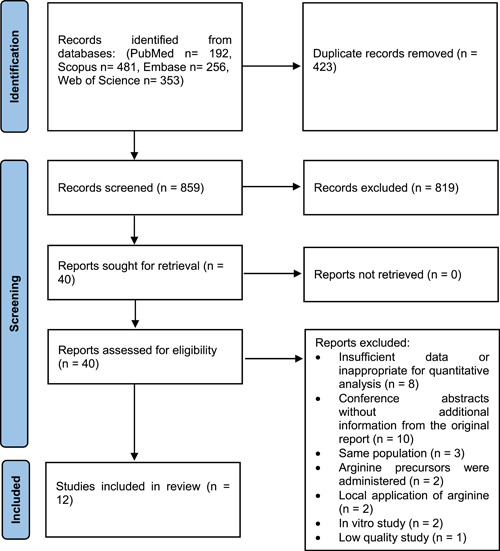
PRISMA 2020 flowchart of study identification, screening, and selection process.

A total of 1282 records were yielded from the systematic search in the four online databases, 192 from PubMed, 481 from Scopus, 256 from Embase, and 353 from Web of Science. After removing 423 duplicate records and 819 records in the first round of screening, 40 records were identified to be evaluated in the second screening and their full texts were retrieved. Interrater agreement of the authors (A. S. and N. D. E.) for screening was 97.1%, with a *Κ* statistic of 71.0% which shows substantial agreement. Among these records, 28 were found to not meet the eligibility criteria and were removed. Despite evaluating the effect of l‐arginine on outcomes of patients with SCD in eight of these removed records, there was insufficient data or heterogenous to each other endpoints which made them inappropriate for quantitative analysis. Other reasons for the exclusion of the records in the second screening along with a full description of the process of selection are depicted in Figure 1.

### 3.2 Characteristics of the included studies

Characteristics of the 12 records in this systematic review and meta‐analysis are provided in Table 1. All the included studies were trials, some of them with a randomized design (six studies). All studies except the one that was conducted by Elias et al.,^8^ were published as an original article; the study by Elias et al. was published as a correspondence. The studies were published between 2003 and 2023. Nigeria with six studies and afterward, the United States with four studies were the main countries in which the studies were conducted. Three hundred and ninety‐nine patients with SCD were assessed in these eligible studies. In six studies, the participants were selected from children and in the other six studies, adult participants were enrolled.

**Table 1:** Characteristics of the studies included in this systematic review and meta‐analysis.

First author	Country	Study design	Sample size (arms of study)	Patients' characteristics (arms of study)	Male (%)	Age (years) Mean (±SD)	HbSS (%)	Intervention type	Duration	Endpoints
Saka et al.^32^	Nigeria	Clinical trial	80 (40/40)	SCA adults in steady state/healthy adults	50	29.4 ± 6.9	100	PO l‐arginine, 1000 mg QD	6 weeks	Fasting blood glucose and insulin, glycated hemoglobin, glucose transporter 1, oxidative stress marker level, plasma amino acid level, plasma NO metabolites
Onalo et al.^10^	Nigeria	RCT	66 (35/31)	SCA children in nonsteady state	57.6	10.6 ± 3.4	100	PO l‐arginine, 100 mg/kg TID	5 days or till discharge	Anthropometric indices, pain severity, plasma aminoacid levels, vital signs
Temiye et al.^9^	Nigeria	Single arm trial	22	SCD children in steady state	NM	5.6 ± 5.1	NM	PO l‐arginine, 500 mg QD	6 weeks	Anthropometric indices, antioxidant enzymes, CBC, LFT, oxidative stress marker level, vital signs
Morris et al.^14^	USA	RCT	12	SCD children in nonsteady state	67	13.6 ± 3	75	Randomized to 1 of 3 IV l‐arginine doses:	7 days or till discharge	Mitochondrial activity and DNA, oxidative stress markers level
								1–100 mg/kg TID		
								2–200 mg/kg as loading and then 100 mg/kg TID		
								3–200 mg/kg as loading and then 300 mg/kg infusion daily		
Eleutério et al.^13^	Brazil	RCT	50 (25/25)	SCA adults in steady state	48	28 ± 9	100	PO l‐arginine, 500 mg QD	4 months	CBC, HbF, hospitalization frequency, plasma NO metabolites, reticulocyte count, pain frequency
Abubakar et al.^31^	Nigeria	RCT	60 (30/30)	SCA children in steady state	NM	7.5 ± 3.9	100	PO l‐arginine, 700–3500 mg BID (based on body weight)	8 weeks	Plasma aminoacid levels, plasma NO metabolites
Jaja et al.^7^	Nigeria	Clinical trial	40 (20/20)	SCA adults in steady state/healthy adults	NM	24.5 ± 5.4	100	PO l‐arginine, 1000 mg QD	6 weeks	Anthropometric indices, CBC, LFT, oxidative stress marker level, plasma amino acid level, plasma NO metabolites
Kehinde et al.^35^	Nigeria	Single arm trial	28	SCA adults in steady state	50	24.5 ± 6.3	100	PO l‐arginine, 1000 mg QD	6 weeks	Antioxidant enzymes, CBC, irreversibly sickled cells percentage, oxidative stress marker level, plasma aminoacid level, osmotic fragility
Morris et al.^34^	USA	RCT	54 (26/28)	SCD children in nonsteady state	48.1	13.9 ± 4	74	PO/IV l‐arginine, 100 mg/kg TID with a maximum dose of 10 g for 15 doses	5 days or till discharge	CBC, hospital length of stay, LFT, pain severity, total opioid use, reticulocyte count
Elias et al.^8^	Brazil	RCT	21 (12/9)	SCA adults in steady state	42.8	NM	100	PO l‐arginine, 250 mg QD	3 months	CBC, HbF, plasma NO metabolites, reticulocyte count
Little et al.^33^	USA	Clinical trial	27 (13/14)	SCA adults in steady state/SCA adults in steady state	40.7	38.4 ± 9.4	100	1‐ PO l‐arginine, 100–200 mg ⁄kg divided TID in one arm2‐ PO sildenafil, 25–100 mg TID in the other arm	12 weeks	Arginase activity, CBC, echocardiographic indices, HbF, 6 min walk test, plasma aminoacid levels, reticulocyte count
Morris et al.^30^	USA	Clinical trial	5	SCA children in steady state	20	16.2 ± 4	100	PO l‐arginine, 100 mg/kg	Single dose	Arginase activity, exhaled NO, plasma aminoacid levels, plasma NO metabolites

Abbreviations: BID, two times a day; CBC, cell blood count; DNA, deoxyribonucleic acid; HbF, fetal hemoglobin; IV, intravenous; LFT, liver function tests; NM, not mentioned; NO, nitric oxide; PO, oral; QD, once daily; RCT, randomized clinical trial; SCA, sickle cell anemia; SCD, sickle cell disease; SD, standard deviation; TID, three times a day.

### 3.3 Pooled effects of l‐arginine consumption

**Figure 2:**
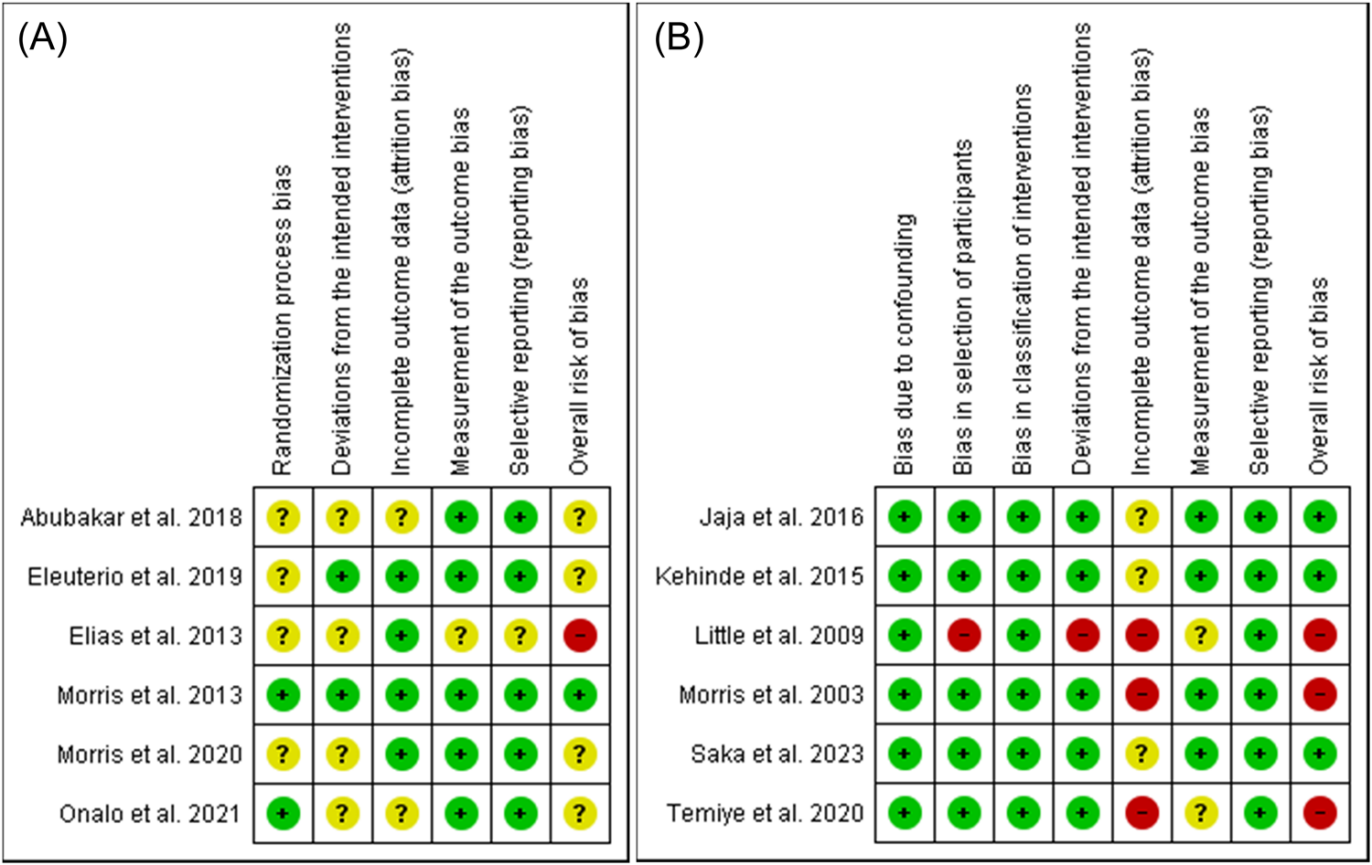
Results of the quality assessment of the included (A) RCT studies (B) non‐RCT studies. RCT, randomized clinical trial.

Figure 3 demonstrates the forest plots for the pooled effects of l‐arginine supplementation on NO metabolites' concentration, hemoglobin, HbF, reticulocyte, malondialdehyde, SBP, DBP, AST, and ALT. Data from six trials were used for the quantitative analysis of changes in the concentration of NO,^7^, ^8^, ^13^, ^30^, ^31^, ^32^ five for changes in hemoglobin,^8^, ^9^, ^33^, ^34^, ^35^ three for changes in HbF,^8^, ^9^, ^33^ three for changes in reticulocytes,^8^, ^33^, ^34^ four for changes in malondialdehyde level,^9^, ^14^, ^32^, ^35^ three for changes in SBP and DBP,^9^, ^10^, ^34^ and three for changes in AST and ALT.^7^, ^9^, ^34^ The results of the quality assessment with ROBINS‐I and RoB2 for included randomized and nonrandomized trials are shown in Figure 2. Among the included studies, four studies had a low risk of bias, four studies had an unclear risk of bias and the other four had a high risk of bias. In each of the outcomes assessed in the study, there was at least one study with a high risk of bias based on the Cochrane tools for risk of bias assessment we used in this review.

The data synthesis showed that l‐arginine consumption significantly increased the level of NO metabolites (Hedge's g: 1.50 with 95% CI of 0.48–1.82, *p* = 0.01, *I* ^2^: 88% with 95% CI of 77%–94%, Figure 3A) and HbF (WMD: 1.69% with 95% CI of 0.86–2.52, *p* = 0.01, *I* ^2^: 0% with 95% CI of 0%–90%, Figure 3C). Besides, it was shown that it significantly decreased SBP (WMD: −8.46 mmHg with 95% CI of −15.58 to −1.33, *p* = 0.04, *I* ^2^: 53% with 95% CI of 0–86, Figure 3F) and AST (Hedge's g: −0.49 with 95% CI of −0.73 to −0.26, *p* = 0.01, *I* ^2^: 0% with 95% CI of 0–90, Figure 3I).

Figure 3The forest plot of individualized and pooled results of l‐arginine supplementation effects on (A) NO metabolites' concentration (calculated as Hedge's g) (B) hemoglobin (g/dL) (C) HbF (%) (D) reticulocyte (calculated as Hedge's g) (E) Malondialdehyde (calculated as Hedge's g) (F) SBP (mmHg) (G) DBP (mmHg) (H) ALT (calculated as Hedge's g) (I) AST (calculated as Hedge's g). ALT, alanine transaminase; AST, aspartate transaminase; DBP, diastolic blood pressure; HbF, hemoglobin F; NO, nitric oxide; SBP, systolic blood pressure.

**Figure:**
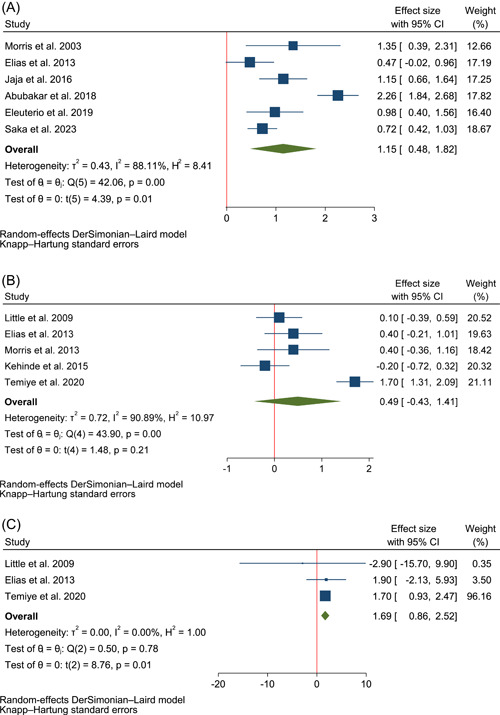

**Figure:**
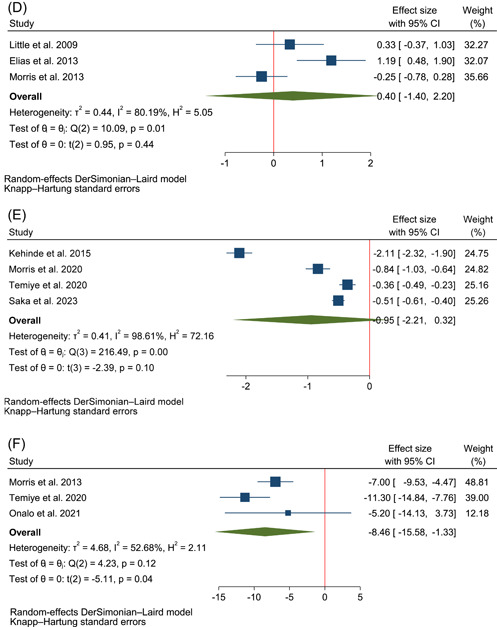

**Figure:**
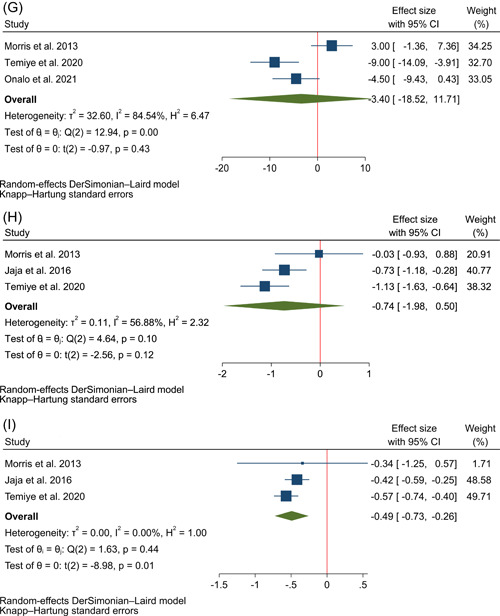

There were no significant changes following administration of l‐arginine in levels of hemoglobin (WMD: 0.49 g/dL with 95% CI of −0.43 to 1.41, *p* = 0.21, *I* ^2^: 91% with 95% CI of 82%–95%, Figure 3B), reticulocytes (Hedge's g: −0.40 with 95% CI of −1.40 to 2.20, *p* = 0.44, *I* ^2^: 80% with 95% CI of 37%–94%, Figure 3D), malondialdehyde (Hedge's g: −0.95 with 95% CI of −2.21 to 0.32, *p* = 0.10, *I* ^2^: 99% with 95% CI of 98–99, Figure 3E), DBP (WMD: −3.40 mmHg with 95% CI of −18.52 to 11.71, *p* = 0.43, *I* ^2^: 85% with 95% CI of 54%–95%, Figure 3G) and ALT (Hedge's g: −0.74 with 95% CI of −1.98 to 0.50, *p* = 0.12, *I* ^2^: 57% with 95% CI of 0–88, Figure 3H).

### 3.4 Additional analyses

As shown in Supporting Information: Figure 1, sensitivity analyses for the changes in the concentrations of NO metabolites, hemoglobin, reticulocyte, malondialdehyde, DBP, and ALT did not result in any significant differences. However, for changes in HbF, SBP, and AST, the pooled effect sizes were sensitive to the “leave‐one‐out” of the studies (Supporting Information: Figure 1). The potential publication bias in this study was assessed using Egger's test. The test showed that the estimation of none of the pooled effect sizes was significantly affected by a considerable publication bias.

Subgroup analysis was done for NO metabolites and hemoglobin based on the age group. The findings of subgroup analysis for NO metabolites accounted for some of the substantial observed heterogeneity, *I* ^2^ of 88.11%, and showed significant increases in the concentration of NO metabolites in both children (two studies, Hedge's g of 1.91 with 95% CI of −3.71 to 7.54, *I* ^2^: 65.52%) and adults (four studies, Hedge's g of 0.81 with 95% CI of 0.36–1.2.5, 30.35%). Similar subgroup analysis for hemoglobin level yielded the same no significant change in the level of hemoglobin following the use of l‐arginine in both children (two studies, WMD of 1.09 with 95% CI of −7.15 to 9.33, *I* ^2^: 88.70%) and adults (three studies, WMD of 0.07 with 95% CI of −0.63 to 0.78, *I* ^2^: 8.29%%).

### 3.5 Certainty of evidence

The certainty of the evidence was very low for four outcomes estimated in our study, change in concentration of NO metabolites, reticulocyte, malondialdehyde, and DBP. The overall certainty for changes in the levels of hemoglobin, HbF, SBP, ALT, and AST was rated as low. Therefore, for none of the outcomes, the certainty of the evidence was moderate or high. The main two reasons for rating down the evidence across different outcomes were the risk of bias and imprecision (Table 2). Besides, there was not any factor to upgrade the quality of the evidence for each of the estimated outcomes.

**Table 2:** Certainty of evidence for each outcome estimated in this systematic review and meta‐analysis using the GRADE approach.

Outcome	Number of participants (number of studies) included for each analysis	Effect size (95% confidence interval)	Quality of evidence	Overall certainty
Nitric oxide metabolites' concentration	196 (6)	1.50 (0.48–1.82)	⊕⊖⊖⊖ Due to risk of bias, indirectness of the evidence and imprecision	Very low
Hemoglobin (g/dl)	138 (5)	0.49 (−0.43 to 1.41)	⊕⊕⊖⊖ Due to risk of bias and imprecision	Low
Fetal hemoglobin (%)	56 (3)	1.69 (0.86–2.52)	⊕⊕⊖⊖ Due to risk of bias and imprecision	Low
Reticulocyte	88 (3)	−0.40 (−1.40 to 2.20)	⊕⊖⊖⊖ Due to risk of bias, imprecision and inconsistency	Very low
Malondialdehyde	102 (4)	−0.95 (−2.21 to 0.32)	⊕⊖⊖⊖ Due to risk of bias, imprecision and inconsistency	Very low
Systolic blood pressure (mmHg)	142 (3)	−8.46 (−15.58 to −1.33)	⊕⊕⊖⊖ Due to risk of bias and imprecision	Low
Diastolic blood pressure (mmHg)	142 (3)	−3.40 (−18.52 to 11.71)	⊕⊖⊖⊖ Due to risk of bias, imprecision and inconsistency	Very low
Alanine transaminase	96 (3)	−0.74 (−1.98 to 0.50)	⊕⊕⊖⊖ Due to risk of bias and imprecision	Low
Aspartate transaminase	96 (3)	−0.49 (−0.73 to −0.26)	⊕⊕⊖⊖ Due to risk of bias and imprecision	Low

### 3.6 Adverse events

Safety data regarding the use of l‐arginine were only pointed to in less than half of the included studies. There were no serious adverse events in the studies which reported this issue.^10^, ^13^, ^14^, ^33^, ^34^ In a 12‐week trial by Little et al., 13 patients received l‐arginine and 14 patients received sildenafil.^33^ In this study, patients in both arms had symptoms of leg edema and ocular complications; however, they were self‐limited and there was no need for a medication change. In Onalo et al. study, one patient had hives during the administration of l‐arginine and that patient was withdrawn from the study.^34^ This patient had a previous history of allergy to paper tape and it was accidentally used; therefore, whether l‐arginine had been involved in the development of the allergic reaction remained unclear.

## 4 DISCUSSION

Our study is the first systematic review and meta‐analysis assessing the effect of the use of l‐arginine in patients with SCD. A total of 12 studies containing detail of 399 patients with SCD were included. Statistical analyses showed there were significant increases in the levels of NO metabolites and HbF following l‐arginine supplementation. Besides, the levels of SBP and AST decreased significantly. However, other study outcomes including hemoglobin, reticulocytes, malondialdehyde, DBP, and ALT did not have significant changes following the use of arginine.

The mechanisms by which the use of l‐arginine could exert these fruitful effects in patients with SCD are mainly attributable to the increased NO concentration. l‐arginine passes through cationic amino acid transporter proteins, especially the cationic amino acid transporter‐1, of the membrane of the endothelial cells and enters the cytoplasm, where the soluble and membrane‐associated NO synthases are predominantly located.^36^, ^37^ The enzymatic activity of NO synthases depends on the availability and the extracellular concentrations of l‐arginine.^38^ NO synthase catalyzes the oxidation of l‐arginine and the production of citrulline and NO. Therefore, the concentration of l‐arginine seems to be tightly related to production and thus, the concentration of NO.^39^ Consistently, our study found a positive association between l‐arginine supplementation and serum nitrogen oxides; There was a statistically significant increase in nitrogen oxides level (Hedge's g of 1.50 with 95% CI of 0.48–1.82) following the use of l‐arginine. In a population‐based study on 2771 individuals, the dietary intake of l‐arginine was measured using a semiquantitative questionnaire.^40^ In this study, it was reported higher intake of l‐arginine was associated with higher concentrations of serum NO compounds. Therefore, not only the use of l‐arginine as a separate medicinal medication or product but also, the intake of diets with higher arginine content might bring the positive effects shown in our study for SCD patients; however, concerning the dysregulated arginine metabolism in these patients, studies are required to assess to whether and to what extent these dietary regimens could help them.

The analyses showed that l‐arginine could lead to a higher level of HbF in patients with SCD. Augmentation of HbF synthesis is among the most effective measures for these patients. In a cohort study on 3764 patients with SCD, risk factors for early death were investigated.^41^ In this study, it was shown that higher levels of HbF were associated with a higher rate of survival. Besides, HbF could help to prevent complications occurring as a result of the sickling phenomenon of altered hemoglobin, successive vaso‐occlusion, and abnormal blood viscosity.^42^, ^43^ The main therapeutic effects of hydroxyurea on morbidity and mortality of patients with SCD are believed to be exerted through its enhancing effect on the level of HbF.^44^ Therefore, l‐arginine supplementation could also result in positive outcomes. The included studies also showed that this effect of l‐arginine on the HbF level could be additive to the effect of hydroxyurea and could further increase the HbF level in those who are on hydroxyurea. In the study conducted by Temiye et al., the study participants were on hydroxyurea therapy before the trial and then, l‐arginine was given to them. The study reported that there was a mean increase of about 32% in the level of HbF in these patients.^9^ A similar finding was observed in Elias et al. work^8^; the participants were taking hydroxyurea for more than 1 year and then they were assigned to either receive l‐arginine or not. After the 12 weeks of trial, the patients in the intervention group had a significant increase in the level of HbF. Therefore, it seems that l‐arginine may have the potential to be used as an adjunct to improve the induction of a high level of HbF.

Significant decreases in levels of AST observed in the study could be indicative of the hepatoprotective effects of l‐arginine. Liver dysfunction is frequently seen in patients with SCD even in the steady state^45^, ^46^, ^47^ and itself, is among the physiopathologic processes for the presentations and the complications of SCD. In a study conducted in Nigeria, 100 participants consisting of 50 patients with SCD and 50 individuals without a history of a medical illness were selected and their liver functions were, then, measured.^48^ The serum concentrations of liver enzymes were shown to be higher in the SCD patients than in the control group. Consistently, in another cross‐sectional study, only one‐quarter of steady‐state patients had normal levels of liver enzymes.^49^ The dysfunction and abnormality in the liver enzymatic activity could further proceed following occlusion of the vessels and sinusoids and subsequent ischemia, and extreme destruction of the red cells with injurious products within a crisis.^50^ Therefore, the hepatoprotective effect of l‐arginine would be of great importance to them. Arginine could also exhibit hepatoprotective properties by several means; reduction of the overall liver tissue inflammation and oxidative stress are the leading modes of action. Studies have shown that l‐arginine could result in a decreased level of different proinflammatory cytokines and nuclear factor‐κB and therefore, downregulates its downstream signaling pathway such as metalloproteinases.^51^, ^52^, ^53^, ^54^

The antioxidant properties of arginine are reported frequently, in both animal experiments and human studies.^55^, ^56^, ^57^, ^58^, ^59^ In a study by Liang et al., male rats were divided into five groups including one negative control group, one positive control with daily vitamin C, and three intervention groups receiving l‐arginine at different doses. The study showed that l‐arginine administration was associated with increased free radical scavenging activity and antioxidant enzymatic activity, comparable to that of vitamin C or even higher for some parameters.^60^ Similarly, in a clinical trial on children with SCD, blood levels of all three measured antioxidant enzymes, catalase, superoxide dismutase, and glutathione peroxidase, increased significantly following 6 weeks of addition of l‐arginine to their therapeutic regimen, and the level of malondialdehyde, an index of lipid peroxidation and presence of oxidative stress, decreased significantly.^9^ This antioxidant effect results from both NO‐independent and dependent mechanisms. Studies have demonstrated that in pathologic conditions such as ischemia, which is among the most common underlying causes in the pathophysiology of SCD's sequels, arginine exhibits preventive effects by acting as a scavenger for oxygen radicals, in particular superoxide ions, the direct/NO independent mechanism.^61^ Besides, with a fair enough amount of arginine, the production of superoxide ions would also diminish. In the latter mechanism, NO, an arginine metabolite, provides the antioxidant effect through various modes of action such as attenuation of the chemistry of the oxidants, augmentation of the potency of antioxidants, and induction of expression of antioxidant enzymes and a series of genes involved in antioxidant homeostasis.^55^, ^62^, ^63^, ^64^ The change in the level of malondialdehyde following supplementation with l‐arginine was, however, not significant in this meta‐analysis. The effect was significant without the Hartung–Knapp adjustment. It is important to note that we were not able to conduct analyses on other parameters of the oxidant–antioxidant system such as serum catalase, glutathione peroxidase, and superoxide dismutase levels due to the limited data. Therefore, by the nonsignificant pooled effect size of l‐arginine on the malondialdehyde system, one could preclude the antioxidant effects on oxidative stress.

This systematic review and meta‐analysis had some limitations. Twelve studies were included in this study but for most of the study outcomes, there were few eligible studies with a rather low number of participants. We used the Hartung–Knapp adjustment due to the small number of included studies. Second, the low number of the included studies for each quantitative analysis limited us in conducting subgroup analyses based on some variables such as the duration of treatment with l‐arginine and the status of the disease activity of the enrolled participants which are of high importance for patients with SCD. Third, although different doses of l‐arginine were given to the participants in the included studies, we were not able to use meta‐regression to explore whether this different dosing could be a source of heterogeneity in the overall estimates. Fourth, we also investigated potential publication bias in this study and the statistical analyses showed no potential publication bias; however, since there was a small number of studies in each analysis, the results of the publication bias test may not be reliable. Fifth, the certainty of the body of evidence for all the outcomes estimated in this study was either low or very low mainly owing to significant limitations in the included studies and imprecision. Therefore, our study showed statistically significant beneficial effects for treatment with l‐arginine without serious adverse effects with low and very low‐quality of evidence. Therefore, its findings should be approached with caution, and well‐developed and adequately powered trials are required to consolidate them.

## 5 CONCLUSION

Our meta‐analysis showed that l‐arginine use for SCD could be beneficial, increase HbF levels, and exert blood pressure‐lowering and hepatoprotective properties. However, for a firm conclusion and rationale for arginine therapy for these patients, more studies with higher population sample sizes, longer follow‐up durations, and well‐developed designs are needed.

## AUTHOR CONTRIBUTIONS

**Alireza Sadeghi**: Conceptualization; formal analysis; investigation; methodology; project administration; software; writing—original draft; writing—review and editing. **Ehsan Taherifard**: Conceptualization; investigation; methodology; project administration; validation; writing—original draft; writing—review and editing. **Niloofar Dehdari Ebrahimi**: Data curation; investigation; methodology; validation; writing—original draft. **Elham Rafiei**: Data curation; investigation; methodology; validation; writing—review & editing **Farshad Hadianfard**: Data curation; investigation; methodology; validation; writing—original draft. **Erfan Taherifard**: Conceptualization; methodology; software; supervision; writing—original draft; writing—review and editing.

## CONFLICT OF INTEREST STATEMENT

The authors declare no conflict of interest.

## TRANSPARENCY STATEMENT

The lead author Erfan Taherifard affirms that this manuscript is an honest, accurate, and transparent account of the study being reported; that no important aspects of the study have been omitted; and that any discrepancies from the study as planned (and, if relevant, registered) have been explained.

## Supporting information

Supporting information.

## Data Availability

The data sets generated during and/or analyzed during the current study are available from the corresponding author on reasonable request.
